# Supplementary material for: Evaluating the Causal Association between Inflammatory Bowel Disease and Risk of Atherosclerotic Cardiovascular Disease: Univariable and Multivariable Mendelian Randomization Study
Source: Biomedicines. 2023 Sep 15;11(9):2543. doi: 10.3390/biomedicines11092543 (PMC10526051; doi:10.3390/biomedicines11092543)
Supplement: Supplementary file 1 [file biomedicines-11-02543-s001.zip › biomedicines-2551568-supplementary.pdf]

Causal association between inflammatory bowel disease and risk of atherosclerotic cardiovascular disease: a Mendelian randomization study

Baike Liu et al.

Supplementary Tables

Table S1. Summary of the genetic instruments identified for IBD, CD, and UC in MR Analyses. 2
Table S2. Statistical power of MR analyses* 3
Table S3. SNPs used as instruments for inflammatory bowel disease, Crohn's disease, and ulcerative colitis. 5
Table S4. Analysis of Heterogeneity and directional pleiotropy. 11
Table S5. Positive and negative control outcome analyses for the relationship of IBD with PSC and Parkinson's disease. 13
Table S6. Description of datasets used for inflammatory markers and common risk factors for ASCVD. 14

Table S1. Summary of the genetic instruments identified for IBD, CD, and UC in MR Analyses.

Exposure	Significant level	No. of SNPs	F statistics (min, max)	Explained variance (R^2)
Inflammatory bowel disease	5.00E-08	115	70.33 (29.86, 500.6)	13.46%
Crohn's disease	5.00E-08	85	78.75 (30.15, 489.58)	16.56%
Ulcerative colitis	5.00E-08	59	69.18 (30.47, 408.13)	8.86%

Table S2. Statistical power of MR analyses*.

Exposre	Outcome	OR = 1.01	OR = 1.02	OR = 1.03	OR = 1.04	OR = 1.05	OR = 1.06	OR = 1.07	OR = 1.1	OR = 1.2	Sample size	Case (%)
IBD	Coronary artery disease (CARDIoGRAMplusC4D plus UKBB + FinnGen)	0.25	0.73	0.97	1	1	1	1	1	1	766,053	20.15%
	Ischemic stroke (MEGASTROKE consortium + FinnGen + UKBB)	0.12	0.35	0.65	0.88	0.97	1	1	1	1	1,020,314	4.71%
	Large artery stroke (MEGASTROKE consortium)	0.06	0.08	0.11	0.16	0.23	0.39	0.67	0.95	1	410,484	1.07%
	Small vessel stroke (MEGASTROKE consortium)	0.06	0.08	0.13	0.19	0.26	0.46	0.75	0.98	1	198,048	2.72%
	Cardioembolic stroke (MEGASTROKE consortium)	0.06	0.09	0.15	0.23	0.34	0.58	0.87	1	1	413,304	1.74%
CD	Coronary artery disease (CARDIoGRAMplusC4D plus UKBB + FinnGen)	0.3	0.81	0.99	1	1	1	1	1	1	766,053	20.15%
	Ischemic stroke (MEGASTROKE consortium + FinnGen + UKBB)	0.14	0.41	0.74	0.94	0.99	1	1	1	1	1,020,314	4.71%

	Large artery stroke (MEGASTROKE consortium)	0.06	0.08	0.13	0.19	0.27	0.47	0.76	0.98	1	410,484	1.07%
	Small vessel stroke (MEGASTROKE consortium)	0.06	0.09	0.14	0.22	0.31	0.54	0.84	0.99	1	198,048	2.72%
	Cardioembolic stroke (MEGASTROKE consortium)	0.06	0.11	0.18	0.28	0.4	0.67	0.93	1	1	413,304	1.74%
UC	Coronary artery disease (CARDIoGRAMplusC4D plus UKBB + FinnGen)	0.18	0.55	0.88	0.99	1	1	1	1	1	766,053	20.15%
	Ischemic stroke (MEGASTROKE consortium + FinnGen + UKBB)	0.1	0.25	0.48	0.72	0.89	0.99	1	1	1	1,020,314	4.71%
	Large artery stroke (MEGASTROKE consortium)	0.05	0.07	0.09	0.12	0.16	0.28	0.5	0.83	0.97	410,484	1.07%
	Small vessel stroke (MEGASTROKE consortium)	0.06	0.07	0.1	0.14	0.19	0.32	0.57	0.9	0.99	198,048	2.72%
	Cardioembolic stroke (MEGASTROKE consortium)	0.06	0.08	0.12	0.17	0.24	0.42	0.7	0.96	1	413,304	1.74%

*statistical power calculation was performed on https://shiny.cnsgenomics.com/mRnd/.

Table S3. SNPs used as instruments for inflammatory bowel disease, Crohn's disease, and ulcerative colitis.

SNP	CHR	EA	OA	BETA	SE	P value	F statistics	R2
IBD								
rs12136659	1	C	T	0.087	0.0142	1.02E-09	37.54	0.00063
rs2488398	1	C	G	0.0985	0.0149	3.63E-11	43.7	0.00073
rs10746475	1	A	T	0.1308	0.0164	1.58E-15	63.61	0.00106
rs112936798	1	C	A	-0.1844	0.0332	2.89E-08	30.85	0.00051
rs35730213	1	C	G	-0.1346	0.014	7.50E-22	92.43	0.00154
rs3024493	1	A	C	0.1911	0.0165	4.04E-31	134.14	0.00223
rs11209013	1	G	A	0.0773	0.0124	4.46E-10	38.86	0.00065
rs11581607	1	A	G	-0.6578	0.0294	4.59E-111	500.6	0.00828
rs1336900	1	A	G	-0.0848	0.0128	2.98E-11	43.89	0.00073
rs10800309	1	G	A	-0.123	0.0133	1.94E-20	85.53	0.00142
rs1268339	1	C	T	0.0907	0.0163	2.75E-08	30.96	0.00052
rs1317209	1	A	G	0.1164	0.016	3.79E-13	52.93	0.00088
rs3820330	1	A	C	-0.0892	0.014	1.72E-10	40.6	0.00068
rs4276914	1	A	G	0.0783	0.0125	3.15E-10	39.24	0.00065
rs7532133	1	G	A	0.0789	0.0134	3.83E-09	34.67	0.00058
rs11677002	2	C	T	-0.0931	0.0126	1.37E-13	54.6	0.00091
rs55946629	2	A	C	0.1298	0.018	5.45E-13	52	0.00087
rs4676408	2	A	G	0.1011	0.013	7.63E-15	60.48	0.00101
rs7608697	2	C	A	0.1395	0.0126	1.67E-28	122.58	0.00204
rs13422838	2	C	T	-0.1143	0.0205	2.56E-08	31.09	0.00052
rs62180107	2	C	G	-0.0797	0.0132	1.55E-09	36.46	0.00061
rs3792111	2	T	C	0.1391	0.0124	5.12E-29	125.84	0.00209
rs1558619	2	T	G	-0.0843	0.0123	8.90E-12	46.97	0.00078
rs76286777	2	C	T	0.0996	0.0151	4.65E-11	43.51	0.00073
rs72852162	2	C	A	-0.1129	0.0202	2.30E-08	31.24	0.00052
rs6740847	2	G	A	-0.0924	0.0125	1.22E-13	54.64	0.00091
rs62183956	2	T	C	-0.078	0.0125	4.49E-10	38.94	0.00065
rs1131095	3	C	T	0.1635	0.0131	1.22E-35	155.77	0.00259
rs56116661	3	T	C	-0.1	0.0163	9.27E-10	37.64	0.00063
rs77272631	3	C	G	0.2293	0.0417	3.72E-08	30.24	0.0005
rs503734	3	G	A	-0.0692	0.0124	2.67E-08	31.14	0.00052
rs2593855	3	T	C	-0.0832	0.014	2.54E-09	35.32	0.00059
rs11734570	4	A	G	0.0694	0.0127	4.80E-08	29.86	0.0005
rs62324212	4	A	C	0.0886	0.0127	2.67E-12	48.67	0.00081
rs4957256	5	T	C	-0.1179	0.0155	3.37E-14	57.86	0.00096
rs17656349	5	T	C	0.0731	0.0125	5.17E-09	34.2	0.00057
rs6579807	5	T	C	0.125	0.0189	4.01E-11	43.74	0.00073
rs1445004	5	T	C	0.1689	0.0127	3.48E-40	176.87	0.00294
rs62378712	5	C	T	-0.0776	0.0142	4.23E-08	29.86	0.0005
rs6873866	5	C	T	-0.0919	0.0128	6.15E-13	51.55	0.00086
rs10041497	5	C	T	0.0819	0.0129	1.95E-10	40.31	0.00067
rs755374	5	T	C	0.1767	0.0134	1.59E-39	173.89	0.00289

rs56235845	5	G	T	0.0877	0.0138	1.77E-10	40.39	0.00067
rs11739135	5	C	G	0.1366	0.0125	1.10E-27	119.42	0.00199
rs341295	5	T	C	0.0702	0.0124	1.45E-08	32.05	0.00053
rs11152949	6	G	A	0.1019	0.0133	1.56E-14	58.7	0.00098
rs1267496	6	C	G	0.1053	0.0159	3.39E-11	43.86	0.00073
rs145568234	6	G	T	0.86	0.0476	4.73E-73	326.42	0.00542
rs6457681	6	T	G	-0.1687	0.0153	3.75E-28	121.58	0.00202
rs4712528	6	C	G	0.1043	0.0152	7.14E-12	47.08	0.00078
rs143210366	6	G	T	0.2836	0.036	3.14E-15	62.06	0.00103
rs62408218	6	T	C	-0.0818	0.0129	2.40E-10	40.21	0.00067
rs212402	6	A	G	-0.0743	0.013	1.06E-08	32.67	0.00054
rs34140409	6	T	C	-0.1583	0.0237	2.28E-11	44.61	0.00074
rs6933404	6	C	T	0.0863	0.0149	6.64E-09	33.55	0.00056
rs35171809	6	G	A	0.1088	0.0123	1.16E-18	78.24	0.0013
rs10953551	7	G	A	-0.1033	0.0127	4.94E-16	66.16	0.0011
rs243505	7	G	A	-0.0805	0.0128	3.04E-10	39.55	0.00066
rs149169037	7	A	G	-0.1338	0.0242	3.26E-08	30.57	0.00051
rs1456896	7	T	C	0.0879	0.0133	4.50E-11	43.68	0.00073
rs62482552	7	A	G	-0.0737	0.0131	1.97E-08	31.65	0.00053
rs11768365	7	G	A	-0.0837	0.0152	3.88E-08	30.32	0.00051
rs78771661	8	T	C	-0.3848	0.0669	8.95E-09	33.08	0.00055
rs4380956	8	A	G	0.0907	0.0127	1.12E-12	51	0.00085
rs938650	8	A	G	-0.1074	0.0189	1.41E-08	32.29	0.00054
rs1887428	9	C	G	-0.1643	0.0131	2.46E-36	157.3	0.00262
rs10114470	9	C	T	0.1475	0.0137	4.10E-27	115.92	0.00193
rs3829110	9	G	A	0.1574	0.0125	3.52E-36	158.56	0.00264
rs1250573	10	A	G	-0.098	0.0138	1.11E-12	50.43	0.00084
rs10826797	10	T	G	-0.099	0.0136	3.99E-13	52.99	0.00088
rs6584282	10	G	A	-0.152	0.0124	1.19E-34	150.26	0.0025
rs11195128	10	T	C	0.0792	0.0133	2.74E-09	35.46	0.00059
rs2384352	10	G	A	0.0951	0.0131	3.12E-13	52.7	0.00088
rs10761659	10	G	A	0.1585	0.0126	2.30E-36	158.24	0.00263
rs7918084	10	T	C	0.071	0.0125	1.38E-08	32.26	0.00054
rs111456533	10	A	G	-0.1031	0.017	1.18E-09	36.78	0.00061
rs11221335	11	C	T	0.0827	0.0148	2.44E-08	31.22	0.00052
rs11236797	11	A	C	0.1488	0.0125	7.19E-33	141.71	0.00236
rs11066188	12	A	G	0.0874	0.013	1.76E-11	45.2	0.00075
rs117981694	12	A	G	0.3452	0.0411	4.53E-17	70.54	0.00118
rs12825700	12	A	G	0.1324	0.0127	1.27E-25	108.68	0.00181
rs3897234	13	C	T	0.0971	0.0145	1.90E-11	44.84	0.00075
rs140933577	13	C	T	-0.1857	0.0305	1.13E-09	37.07	0.00062
rs194746	14	T	C	0.0833	0.0124	1.84E-11	45.13	0.00075
rs3850378	14	C	T	0.1536	0.0207	1.10E-13	55.06	0.00092
rs1864239	15	G	A	1.3366	0.1782	6.27E-14	56.26	0.00094
rs56062135	15	T	C	0.1382	0.0145	1.37E-21	90.84	0.00151
rs7190426	16	C	A	-0.0872	0.0155	2.06E-08	31.65	0.00053

rs28374519	16	A	G	-0.1105	0.0137	6.55E-16	65.06	0.00108
rs9934775	16	T	C	-0.1116	0.0172	8.77E-11	42.1	0.0007
rs8056255	16	A	T	0.2765	0.0327	2.99E-17	71.5	0.00119
rs11548656	16	G	A	-0.2374	0.0362	5.18E-11	43.01	0.00072
rs749910	16	A	G	0.1961	0.0138	7.83E-46	201.93	0.00336
rs2301127	16	A	G	0.0783	0.0126	4.96E-10	38.62	0.00064
rs16940202	16	C	T	0.113	0.0169	2.50E-11	44.71	0.00075
rs12936409	17	T	C	0.1406	0.0124	7.73E-30	128.57	0.00214
rs744166	17	G	A	-0.1109	0.0126	1.34E-18	77.47	0.00129
rs714910	17	C	A	-0.0959	0.0139	6.23E-12	47.6	0.00079
rs1319951	18	G	C	-0.0851	0.0147	7.50E-09	33.51	0.00056
rs80262450	18	A	G	0.1581	0.019	1.04E-16	69.24	0.00115
rs4807569	19	C	A	0.1281	0.0152	4.24E-17	71.02	0.00118
rs7256518	19	A	G	-0.1665	0.0276	1.63E-09	36.39	0.00061
rs62126610	19	G	A	0.1407	0.0166	2.60E-17	71.84	0.0012
rs11669299	19	T	C	-0.1107	0.0157	1.84E-12	49.72	0.00083
rs6062496	20	A	G	0.137	0.0129	2.83E-26	112.79	0.00188
rs4256018	20	G	T	0.0786	0.0138	1.23E-08	32.44	0.00054
rs6017342	20	C	A	0.1156	0.0135	1.07E-17	73.32	0.00122
rs6063502	20	G	A	-0.0734	0.0134	4.55E-08	30	0.0005
rs154873	20	A	G	-0.0813	0.0132	7.38E-10	37.93	0.00063
rs1297264	21	G	A	-0.1462	0.0126	3.98E-31	134.63	0.00224
rs2836881	21	T	G	-0.1643	0.0146	1.96E-29	126.64	0.00211
rs2838517	21	C	T	-0.128	0.0125	1.83E-24	104.86	0.00175
rs2413583	22	T	C	-0.1732	0.0171	4.60E-24	102.59	0.00171
rs5754100	22	C	T	0.1293	0.016	7.14E-16	65.31	0.00109
rs5763793	22	T	G	0.0734	0.013	1.47E-08	31.88	0.00053
CD								
rs12131079	1	T	C	-0.1088	0.0174	3.99E-10	39.1	0.00097
rs35730213	1	C	G	-0.1166	0.0181	1.17E-10	41.5	0.00103
rs3122605	1	A	G	-0.1748	0.0227	1.24E-14	59.3	0.00147
rs114802258	1	T	C	-0.2245	0.0384	5.11E-09	34.18	0.00085
rs4316387	1	C	T	-0.1292	0.0189	7.74E-12	46.73	0.00116
rs6679677	1	A	C	-0.2275	0.0286	1.77E-15	63.27	0.00157
rs6704109	1	T	C	0.1748	0.0181	5.10E-22	93.27	0.00231
rs7517847	1	G	T	-0.3447	0.0165	5.84E-97	436.43	0.01072
rs11683692	2	C	T	-0.2144	0.038	1.75E-08	31.83	0.00079
rs4343432	2	G	A	0.1123	0.0162	3.50E-12	48.05	0.00119
rs11677002	2	C	T	-0.1124	0.0163	4.57E-12	47.55	0.00118
rs34004493	2	G	A	0.1258	0.0179	2.00E-12	49.39	0.00123
rs3816234	2	A	G	0.2704	0.0162	1.51E-62	278.6	0.00687
rs55946629	2	A	C	0.1755	0.0231	2.85E-14	57.72	0.00143
rs7608697	2	C	A	0.1229	0.0163	4.03E-14	56.85	0.00141
rs6740847	2	G	A	-0.104	0.0161	9.72E-11	41.73	0.00104
rs1583792	2	T	C	-0.0882	0.016	3.26E-08	30.39	0.00075
rs56116661	3	T	C	-0.1312	0.0212	5.67E-10	38.3	0.00095

rs6808936	3	G	A	0.0904	0.0161	1.93E-08	31.53	0.00078
rs9836291	3	A	G	0.1722	0.017	3.77E-24	102.6	0.00254
rs2581828	3	G	C	-0.0941	0.0162	6.46E-09	33.74	0.00084
rs73243877	4	G	A	0.1164	0.0212	4.12E-08	30.15	0.00075
rs13107325	4	T	C	0.2006	0.0284	1.66E-12	49.89	0.00124
rs62324212	4	A	C	0.106	0.0163	8.02E-11	42.29	0.00105
rs6579807	5	T	C	0.1993	0.0244	3.44E-16	66.72	0.00165
rs755374	5	T	C	0.1969	0.0174	1.38E-29	128.05	0.00317
rs6451494	5	C	T	0.2605	0.0166	8.26E-56	246.26	0.00608
rs112856973	5	C	T	-0.1612	0.0243	3.61E-11	44.01	0.00109
rs6873866	5	C	T	-0.1314	0.0164	1.35E-15	64.2	0.00159
rs2188962	5	T	C	0.2004	0.016	5.59E-36	156.88	0.00388
rs181826	5	A	C	0.1162	0.0167	3.24E-12	48.41	0.0012
rs1012636	6	T	G	0.1291	0.0198	7.01E-11	42.51	0.00105
rs1321859	6	T	C	-0.1049	0.0172	1.18E-09	37.2	0.00092
rs73516754	6	C	A	0.1423	0.0169	4.04E-17	70.9	0.00176
rs35171809	6	G	A	0.1566	0.0159	9.07E-23	97	0.0024
rs111281598	6	C	T	0.2745	0.0316	4.17E-18	75.46	0.00187
rs7753014	6	G	C	-0.0989	0.0163	1.39E-09	36.81	0.00091
rs145568234	6	G	T	0.8602	0.0633	4.31E-42	184.67	0.00457
rs9482770	6	C	T	0.0987	0.0162	1.01E-09	37.12	0.00092
rs9501641	6	T	C	0.3027	0.0432	2.57E-12	49.1	0.00122
rs212409	6	A	G	-0.1096	0.0162	1.49E-11	45.77	0.00114
rs9656588	7	C	T	0.1183	0.0173	8.73E-12	46.76	0.00116
rs938650	8	A	G	-0.1747	0.0247	1.65E-12	50.03	0.00124
rs4380956	8	A	G	0.132	0.0165	1.15E-15	64	0.00159
rs79832570	8	C	T	0.2234	0.0344	8.90E-11	42.17	0.00105
rs10114470	9	C	T	0.1687	0.0177	1.76E-21	90.84	0.00225
rs1887428	9	C	G	-0.166	0.0169	8.54E-23	96.48	0.00239
rs4077515	9	T	C	0.1848	0.0162	3.14E-30	130.13	0.00322
rs10884966	10	A	G	0.1131	0.0171	4.13E-11	43.75	0.00109
rs61839660	10	T	C	0.1468	0.0261	1.98E-08	31.64	0.00079
rs2002695	10	G	A	-0.1293	0.0189	8.31E-12	46.8	0.00116
rs10822050	10	C	T	0.1827	0.0162	2.35E-29	127.19	0.00315
rs2675670	10	C	G	0.1074	0.0161	2.89E-11	44.5	0.0011
rs1148246	10	T	C	-0.1323	0.0167	2.09E-15	62.76	0.00156
rs1250573	10	A	G	-0.1522	0.0179	1.92E-17	72.3	0.00179
rs6584282	10	G	A	-0.1658	0.016	3.44E-25	107.38	0.00266
rs11236797	11	A	C	0.176	0.0161	8.51E-28	119.5	0.00296
rs28999107	12	T	G	0.1083	0.0178	1.06E-09	37.02	0.00092
rs77566919	12	A	G	-0.1089	0.0185	4.13E-09	34.65	0.00086
rs34635748	12	T	C	0.4794	0.0504	1.95E-21	90.48	0.00224
rs1373904	13	G	A	0.141	0.0189	9.11E-14	55.66	0.00138
rs194746	14	T	C	0.0975	0.0161	1.24E-09	36.67	0.00091
rs3850378	14	C	T	0.199	0.0267	8.31E-14	55.55	0.00138
rs72743461	15	A	C	0.1684	0.0187	2.26E-19	81.1	0.00201

rs2021511	16	T	C	-0.1082	0.0182	2.63E-09	35.34	0.00088
rs42861	16	G	A	0.1243	0.0167	8.87E-14	55.4	0.00137
rs2076756	16	G	A	0.385	0.0174	1.80E-108	489.58	0.01201
rs7195228	16	G	C	-0.1327	0.0209	2.09E-10	40.31	0.001
rs72798422	16	C	T	0.5495	0.0382	6.05E-47	206.92	0.00511
rs2948542	17	G	A	0.1016	0.0163	5.15E-10	38.85	0.00096
rs714910	17	C	A	-0.1531	0.0181	2.49E-17	71.55	0.00177
rs12936409	17	T	C	0.1426	0.016	4.31E-19	79.43	0.00197
rs744166	17	G	A	-0.1142	0.0162	1.80E-12	49.69	0.00123
rs80262450	18	A	G	0.2268	0.0244	1.34E-20	86.4	0.00214
rs144309607	19	T	C	-0.3712	0.047	2.69E-15	62.38	0.00155
rs62126620	19	A	G	0.144	0.0201	8.61E-13	51.33	0.00127
rs4807570	19	A	G	0.1811	0.0193	6.03E-21	88.05	0.00218
rs492602	19	G	A	0.1084	0.0162	2.33E-11	44.77	0.00111
rs6062496	20	A	G	0.1223	0.0167	2.62E-13	53.63	0.00133
rs3761158	20	A	G	-0.1098	0.0165	2.65E-11	44.28	0.0011
rs1297264	21	G	A	-0.1769	0.0163	1.59E-27	117.78	0.00292
rs2284553	21	G	A	0.1277	0.0165	1.14E-14	59.9	0.00149
rs2838517	21	C	T	-0.1456	0.0162	2.03E-19	80.78	0.002
rs2143178	22	C	T	-0.2087	0.0223	6.84E-21	87.59	0.00217
rs5754100	22	C	T	0.1687	0.0206	3.02E-16	67.06	0.00166
UC								
rs7544646	1	G	C	-0.1168	0.016	2.53E-13	53.29	0.00116
rs3024493	1	A	C	0.21	0.0209	7.46E-24	100.96	0.00219
rs3820330	1	A	C	-0.1587	0.0178	3.91E-19	79.49	0.00173
rs11209026	1	A	G	-0.483	0.0358	1.99E-41	182.02	0.00394
rs6658353	1	C	G	-0.1569	0.016	1.17E-22	96.16	0.00209
rs7554511	1	A	C	-0.1448	0.0178	4.27E-16	66.18	0.00144
rs7523335	1	A	G	-0.1389	0.021	3.42E-11	43.75	0.00095
rs2816954	1	A	T	0.1375	0.0229	1.80E-09	36.05	0.00078
rs1317209	1	A	G	0.1818	0.0203	2.90E-19	80.2	0.00174
rs79051659	1	A	G	0.1605	0.0264	1.30E-09	36.96	0.0008
rs7608697	2	C	A	0.1597	0.0161	3.03E-23	98.39	0.00214
rs55905347	2	A	G	0.1054	0.0166	2.09E-10	40.31	0.00088
rs62180181	2	T	C	0.1226	0.0171	8.08E-13	51.4	0.00112
rs4676408	2	A	G	0.1433	0.0167	1.19E-17	73.63	0.0016
rs1811711	2	G	C	-0.1299	0.0223	6.09E-09	33.93	0.00074
rs1131095	3	C	T	0.1593	0.0168	2.18E-21	89.91	0.00195
rs755374	5	T	C	0.1714	0.0171	9.73E-24	100.47	0.00218
rs72704802	5	T	C	-0.1223	0.0206	2.89E-09	35.25	0.00077
rs17715902	5	A	G	0.0974	0.0166	4.62E-09	34.43	0.00075
rs6889364	5	A	G	0.1318	0.0228	7.87E-09	33.42	0.00073
rs17656349	5	T	C	0.09	0.0159	1.54E-08	32.04	0.0007
rs67111717	5	G	A	0.0944	0.0171	3.27E-08	30.48	0.00066
rs9267798	6	C	G	0.2486	0.028	6.54E-19	78.83	0.00171
rs9271176	6	G	A	-0.3495	0.0173	4.20E-91	408.13	0.0088

rs28383224	6	G	A	-0.1468	0.0165	4.65E-19	79.16	0.00172
rs3734851	6	A	G	0.5033	0.0584	6.58E-18	74.27	0.00161
rs13200059	6	A	G	0.2944	0.0436	1.48E-11	45.59	0.00099
rs6933404	6	C	T	0.1486	0.0188	2.69E-15	62.48	0.00136
rs113986290	6	T	C	-0.3066	0.0531	7.59E-09	33.34	0.00072
rs798506	7	C	T	-0.1206	0.0179	1.47E-11	45.39	0.00099
rs4728142	7	A	G	0.0995	0.0158	3.23E-10	39.66	0.00086
rs989960	7	T	C	-0.1214	0.016	3.28E-14	57.57	0.00125
rs10272963	7	T	C	-0.1512	0.016	4.11E-21	89.3	0.00194
rs1887428	9	C	G	-0.167	0.0166	9.65E-24	101.21	0.0022
rs10817678	9	A	G	0.1332	0.017	4.42E-15	61.39	0.00133
rs3812565	9	C	T	0.1335	0.016	6.50E-17	69.62	0.00151
rs10761659	10	G	A	0.1276	0.016	1.33E-15	63.6	0.00138
rs7911117	10	G	T	-0.1342	0.0239	1.84E-08	31.53	0.00069
rs7911680	10	C	A	-0.1525	0.0159	6.71E-22	91.99	0.002
rs2212434	11	T	C	0.1252	0.0159	2.80E-15	62	0.00135
rs2045241	11	A	G	-0.1063	0.0169	2.83E-10	39.56	0.00086
rs12825700	12	A	G	0.1889	0.0161	7.33E-32	137.66	0.00299
rs1359946	13	A	G	0.1571	0.0202	6.58E-15	60.49	0.00131
rs56062135	15	T	C	0.1078	0.0184	4.66E-09	34.32	0.00075
rs11645239	16	G	C	-0.1174	0.02	4.14E-09	34.46	0.00075
rs7203363	16	A	T	0.1071	0.0189	1.41E-08	32.11	0.0007
rs16940186	16	C	T	0.1357	0.0214	2.18E-10	40.21	0.00087
rs12936409	17	T	C	0.1365	0.0158	5.62E-18	74.64	0.00162
rs11651246	17	G	T	0.147	0.0219	2.01E-11	45.06	0.00098
rs10408351	19	A	G	0.1548	0.0204	2.92E-14	57.58	0.00125
rs78064630	19	A	G	0.1759	0.0308	1.08E-08	32.62	0.00071
rs6062496	20	A	G	0.1359	0.0163	8.97E-17	69.51	0.00151
rs6017342	20	C	A	0.1944	0.017	3.95E-30	130.77	0.00284
rs2836881	21	T	G	-0.2217	0.0186	1.11E-32	142.07	0.00308
rs2838517	21	C	T	-0.1177	0.016	1.78E-13	54.11	0.00118
rs1736161	21	A	G	-0.1227	0.0161	2.22E-14	58.08	0.00126
rs9611131	22	C	T	-0.1494	0.0227	5.11E-11	43.32	0.00094
rs4993442	22	T	G	-0.0988	0.0179	3.54E-08	30.47	0.00066
rs137845	22	G	A	0.1011	0.0158	1.50E-10	40.94	0.00089

CHR, chromosome; EA, effect allele; OA, other allele; EAF, effect allele frequency; SE, standard error.

Table S4. Analysis of Heterogeneity and directional pleiotropy.

Exposure	Outcome	Heterogeneity			MR-Egger test for directional pleiotropy		
		Q	df	p-value	Intercept	se	p-value
IBD	Coronary artery disease (CARDIoGRAMplusC4D plus UKBB)	274.169	99	2.28E-18	0.593	0.491	0.230
	Coronary artery disease (FinnGen)	184.148	100	6.18E-07	0.174	0.260	0.504
	Ischemic stroke (MEGASTROKE consortium)	170.847	102	2.31E-05	0.315	0.363	0.388
	Ischemic stroke (FinnGen)	118.225	100	0.103	-0.246	0.208	0.240
	Ischemic stroke (UKBB)	129.587	102	0.034	0.399	0.309	0.199
	Large artery stroke (MEGASTROKE consortium)	161.883	102	1.48E-04	-0.223	0.360	0.537
	Small vessel stroke (MEGASTROKE consortium)	109.572	102	0.286	0.372	0.290	0.202
	Cardioembolic stroke (MEGASTROKE consortium)	147.405	102	0.002	0.311	0.340	0.362
CD	Coronary artery disease (CARDIoGRAMplusC4D plus UKBB)	227.942	75	2.44E-17	0.389	0.600	0.519
	Coronary artery disease (FinnGen)	126.304	77	3.40E-04	-0.128	0.429	0.766
	Ischemic stroke (MEGASTROKE consortium)	97.708	78	0.065	0.399	0.362	0.274
	Ischemic stroke (FinnGen)	102.000	77	0.030	0.446	0.382	0.246
	Ischemic stroke (UKBB)	88.881	77	0.167	-0.075	0.361	0.837
	Large artery stroke (MEGASTROKE consortium)	85.629	78	0.259	0.373	0.341	0.278
	Small vessel stroke (MEGASTROKE consortium)	66.888	78	0.811	0.334	0.304	0.276
	Cardioembolic stroke (MEGASTROKE consortium)	77.404	78	0.498	0.354	0.324	0.278
UC	Coronary artery disease (CARDIoGRAMplusC4D plus UKBB)	144.303	50	4.38E-11	0.282	0.764	0.714
	Coronary artery disease (FinnGen)	96.119	49	6.71E-05	0.479	0.661	0.473
	Ischemic stroke (MEGASTROKE consortium)	49.096	50	0.510	0.640	0.460	0.170
	Ischemic stroke (FinnGen)	45.681	49	0.608	0.668	0.452	0.146
	Ischemic stroke (UKBB)	66.382	50	0.060	0.301	0.514	0.560

Large artery stroke (MEGASTROKE consortium)	68.246	50	0.044	0.259	0.545	0.636
Small vessel stroke (MEGASTROKE consortium)	59.533	50	0.167	0.737	0.495	0.143
Cardioembolic stroke (MEGASTROKE consortium)	55.319	50	0.281	0.25	0.481	0.605

UKBB, UK Biobank; Q, Cochran's Q statistics; df, degree of freedom; se, standard error

Table S5. Positive and negative control outcome analyses for the relationship of IBD with PSC and Parkinson's disease.

Outcome	Method	OR (95%CI)	p-value
PSC	Inverse variance weighted	1.511 (1.307 to 1.747)	2.39E-08
	MR Egger	1.632 (1.139 to 2.338)	9.19E-03
	Weighted median	1.253 (1.108 to 1.417)	3.18E-04
	MR-RAPS	1.339 (1.194 to 1.502)	6.11E-07
	MR-PRESSO	1.318 (1.21 to 1.436)	1.56E-08
Parkinson's disease	Inverse variance weighted	1.017 (0.98 to 1.055)	0.383
	MR Egger	1.045 (0.959 to 1.14)	0.319
	Weighted median	1.012 (0.956 to 1.07)	0.681
	MR-RAPS	1.022 (0.984 to 1.061)	0.259
	MR-PRESSO	1.007 (0.976 to 1.04)	0.653

PSC, primary sclerosing cholangitis; MR-RAPS, MR Robust Adjusted Profile Score; MR-PRESSO, MR pleiotropy residual sum and outlier.

Table S6. Description of datasets used for inflammatory markers and common risk factors for ASCVD.

Phenotype	Data sources	No. of Cases	No. of Controls	Sample size	Population	PMID
		**Inflammatory markers**				
TNF-α	Ari V Ahola-Olli et al.	-	-	3,454	European	27989323
CRP	Symen Ligthart et al.	-	-	204,402	European	30388399
		**Common risk factors for ASCVD**				
BMI	GIANT consortium	-	-	681,275	European	30124842
Smoking initiation	Mengzhen Liu et al.	311,629	321,173	607,291	European	30643251
Alcoholic drinks per week	Mengzhen Liu et al.	-	-	335,394	European	30643251
Type 2 diabetes	DIAGRAM consortium	10,247	53,924	64,171	European	22885922

ASCVD, atherosclerotic cardiovascular disease; TNF-α, Tumor necrosis factor alpha; CRP, C-reactive protein; BMI, body mass index
